# Ecological grief in the Fernald Community Cohort: A pilot study

**DOI:** 10.1371/journal.pmen.0000049

**Published:** 2024-06-18

**Authors:** Sarah Fitzpatrick, Rachael D. Nolan, Jeffrey S. Hallam, Susan M. Pinney

**Affiliations:** 1 Department of Environmental and Public Health Sciences, College of Medicine, University of Cincinnati, Cincinnati, Ohio, United States of America; 2 Colorado Department of Public Health & Environment, Air Pollution Control Division, Denver, Colorado, United States of America; 3 College of Public Health, Kent State University, Kent, Ohio, United States of America; 4 Healthy Communities Research Institute, Kent State University, Kent, Ohio, United States of America; Sigmund Freud University Vienna, AUSTRIA

## Abstract

Ecological grief is a psychological response to environmental loss. Ecological grief is especially pronounced in people with close relationships with the environment. The Fernald Community Cohort (FCC) included people who lived near a uranium processing site, also known as the Feed Material Production Center (FMPC), in Fernald, Ohio. The FMPC leaked contaminants such as uranium into the environment, consequently threatening the health, safety, and long-term emotional well-being of people who lived nearby. This study aimed to examine the degree of ecological grief reported by the FCC participants and to discern their degree of ecological grief by residential proximity (how close they lived) to the FMPC, estimated uranium exposure, and cancer diagnosis. Participants were invited to complete a questionnaire to assess their degree of ecological grief. Other variables were collected from the existing FCC database. Ecological grief was prevalent among 55.3% of respondents. A direct relationship between ecological grief, residential proximity, and cancer diagnosis was found, with an indirect relationship between ecological grief and uranium exposure. The strongest predictor of ecological grief was a cancer diagnosis. The findings warrant future studies to mitigate the psychological impact of environmental loss on FCC participants.

## Introduction

Ecological disasters are large-scale catastrophic events that cause widespread destruction or distress. Fires, explosions, chemical or radioactive releases from industrial point sources, extreme weather events, natural disasters, and climate change can cause them. Ecological disasters can occur abruptly or gradually. In many cases where environmental contamination occurs, the process ensues over an extended period. Often, disasters affect livelihoods and social processes, which can then cause disruptions to services, social networks, and communal loss of resources. Additionally, it is common for humans to face a threat to physical and mental health, experience consequential trauma, and endure the destruction or loss of valued landscapes, species, ecosystems, and property [1].

Ecological grief is an emerging topic in the literature, which has been defined as a natural human psychological response to environmental loss or destruction, including the loss or destruction of valued landscapes, ecosystems, and species. Environmental losses associated with ecological grief can be acute, chronic, or cumulative and can be caused by natural and manufactured events [2]. Place attachment, or the emotional bond between a person and a place, is theorized to be the framework behind ecological grief [3]. The current literature suggests that many people are attached to a place and construct a part of their identity around it. When that valued place is altered or destroyed, it has the potential to disrupt their self-identity, resulting in ecological grief [3]. Ecological grief is associated with a wide variety of intense emotional reactions, including hopelessness, guilt, denial, depression, despair, frustration, anger, fear, shock, bitterness, loss of place, inability to concentrate, lowered self-worth, emptiness in life, strong yearning, and disorientation [3–11]. Psychosocial responses to ecological grief identified in the current literature include post-traumatic stress disorder, depression, anxiety, chronic illness, drug and alcohol abuse, sleep disorders, suicidal ideation, and impaired physical, psychological, and social functioning [4,6,9,11–13]. Stress accumulated from ecological grief can also amplify other stressors, such as mental disorders, economic hardships, and somatic illness [3,6,7]. Recent research on ecological grief has suggested that manufactured disasters have a more "profound effect on victims than natural disasters" [7]. This effect is thought to be due to the simple difference between natural and manufactured disasters, with the first being caused by natural forces and the latter being caused by human activity. Human-induced disasters can then be blamed on human activity and may be perceived as preventable.

In contrast, natural disasters cannot be as easily controlled or prevented and occur without human involvement. Ecological grief can be felt universally, even years after an ecological loss event occurs. The prevalence of ecological grief is especially pronounced in people with close living, cultural, or working relationships with the natural world, including, but not limited to, indigenous peoples, farming communities, and scientists [3,5,8,12].

The Fernald Superfund site, near Cincinnati, Ohio, is a former United States Department of Energy (USDOE) nuclear fuel processing plant known for historically causing industrial environmental contamination. In the early 1950s, the USDOE built the Feed Material Production Center (FMPC) in a rural farming community in Fernald, Ohio. The FMPC served as a uranium refinery from 1951 to 1989, and chemical releases from the FMPC site resulted in environmental contamination of radon and uranium, in addition to various other organic and inorganic chemicals. The air, water, and soil were all contaminated, consequently threatening the health, safety, and long-term emotional well-being of people living or working on or in close proximity to the site [14]. To mitigate these effects, the USDOE settled litigation with the Fernald community, resulting in a $78 million award to the citizens for physical and emotional harm, loss of property value, and an 18-year medical surveillance program. The Fernald Medical Monitoring Program (FMMP) lasted from 1990 to 2008 and included 9,782 participants [14]. Funding for the physical examinations and testing component of the FMMP ended in November 2008, but the data and specimens collected during the FMMP continue to be available for research. In 2010, the Special Master of the Fernald Settlement Fund agreed to transfer the FMMP data and biospecimen collection to the University of Cincinnati to maintain the database and biospecimen collection for research. The Fernald Medical Monitoring Program became the Fernald Community Cohort (FCC) [15]. The community, to the present day, continues to endure the physical and mental health impacts of this industrial disaster [15,16]. Adverse human health effects caused by exposure to the FMPC’s environmental uranium contamination, such as cancer, were directly observed in the FMMP and the FCC [15]. The environmental loss also impacted the community’s financial stability from decreased property value and degradation of the land, in addition to the loss of livelihood for the farming community.

Given the widespread emotional distress and grief associated with environmental loss, in addition to the impact of environmental contamination on physical health (e.g., cancer), the FCC presents a unique opportunity to study ecological grief in a community affected by environmental radiation contamination. The specific aims of this cross-sectional pilot study were to 1) examine the degree of ecological grief reported by FCC participants; 2) discern the degree of ecological grief among FCC participants by proximity to the plant; 3) assess the degree of ecological grief among FCC participants by cancer diagnosis; and 4) assess the degree of ecological grief among FCC participants by level of uranium exposure. It was hypothesized that ecological grief would be prevalent among FCC participants. The findings from this research can be used to warrant future studies on the impact of ecological grief among FCC participants and to inform future intervention development to mitigate the psychological impact of environmental and health loss at contamination sites.

## Materials and methods

This study utilized data, which the University of Cincinnati Medical IRB approved in 2012, Protocol 2012–3745. The ClinicalTrials.gov identifier is NCT02295085. Participants eligible for this research study included those enrolled in the FCC. The FCC inclusion and exclusion criteria are outlined below and were obtained from ClinicalTrials.gov.

### Inclusion criteria

Lived or worked within five miles of the FMPC for two consecutive years between January 1, 1952, and December 18, 1984.

### Exclusion criteria

The sample did not include anyone who worked at the FMPC or was employed by the US Department of Energy or National Lead of Ohio, Inc. (Plant Contractor).

### Study procedures

As part of the FMMP, participants were provided extensive medical testing, including physical examinations, diagnostic testing, and questionnaires. Questionnaires were completed annually throughout the FMMP, and reexaminations were offered every 2–3 years. All participants of the FMMP signed consent forms to use their biospecimens and data for future research. More detailed information about recruitment methods and study procedures for the FMMP can be found elsewhere [15–17]. Data collected from the FCC was utilized in this study for demographic variables such as age, sex, education level, cancer diagnosis, level of uranium exposure, and proximity to the FMPC. Individual levels of uranium exposure were calculated using algorithms developed by the CDC Fernald Dosimetry Reconstruction Project. As part of the ongoing FCC surveying, in 2020, participants were invited to complete a four-item questionnaire to assess ecological grief’s impact among FCC participants, shown in Table 1. The instrument was adapted Adapted from the Texas Revised Inventory of Grief [18]. Participants of the FCC were sent this ecological grief questionnaire via the United States Postal Service and online. Participants were recruited to this research study on a volunteer basis and made aware that they could withdraw from the study at any time. After data collection was complete and prior to data analysis, the data were de-identified.

**Table 1:** Ecological grief questionnaire.

Directions: Please circle the number (1–5) that best reflects your agreement with each of the following statements.		Strongly Disagree	Disagree	Neither Agree nor Disagree	Agree	Strongly Agree
1.	I still get upset when I think about the contamination at Fernald Preserve.	1	2	3	4	5
2.	Things and people around me are still affected by what happened at Fernald Preserve.	1	2	3	4	5
3.	I believe that the contamination at Fernald Preserve is responsible for the loss (e.g., health, safety, trust) I have experienced.	1	2	3	4	5
4.	I am preoccupied with thoughts about the contamination at Fernald Preserve.	1	2	3	4	5

### Statistical analysis

Descriptive statistics, including means, standard deviations, and frequencies depending on the level of data, were calculated for demographic and main study variables using IBM SPSS (v. 28) [19]. A preliminary analysis was undertaken to assess the psychometric properties of the ecological grief scale. Before the path analysis, data were assessed using Weston and Gore’s criteria for univariate and multivariate normality, linearity, and homoscedasticity [20]. Data were screened for outliers, and variables were evaluated for multicollinearity and singularity. There were no missing data. The sample had sufficient statistical power to detect a significant relationship between uranium exposure, cancer diagnosis, and ecological grief. Structural equation modeling methods were used in the path analysis so that every path could be modeled at one time, accounting for the variance of each association. Paths were specified from proximity and uranium concentration onto ecological grief, proximity onto ecological grief, and proximity and uranium concentration and cancer diagnosis onto ecological grief. Baseline values were linked to each variable, and results were presented as standardized path coefficients for direct comparison. The path analyses were performed using IBM Amos (v. 28) [21]. All analytic assumptions were verified, with the theta parameterization being utilized due to the categorical nature of ecological grief and cancer diagnosis.

## Results

The Ecological Grief Scale analysis showed a mean score of 13.31 (SD 3.75) with a possible minimum score of 4 and a maximum score of 20. Cronbach’s alpha for the scale was .85. Scores on the Ecological Grief Scale were categorized based on percentiles (25^th^, 50^th^, and 75^th^) with a low level of grief scoring 4–11, moderate grief scoring 12–15, and severe grief scoring 16–20. In this sample, 27% reported low grief, 44% reported moderate, and 29% reported severe (Table 2). All participants resided within five miles of the FMPC; on average, residential proximity from the FMPC was 3.04 miles (SD = 1.28). About 18% (n = 672) of the sample reported a cancer diagnosis. About half (46.3%; n = 1,646) of the sample were found to have medium (18.6%) to high (27.7%) uranium exposure based on the biomarkers present in the blood samples obtained during the FMMP. In the sample, 57.6% of the respondents identified as female, and 42.4% of the sample identified as male. The age range was 38 to 100, with an average of 66.5 years old (SD = 12.61). Additionally, 5.4% of respondents had some high school education, 27.7% were only high school graduates, and 65.7% had some college or college graduates.

**Table 2:** Ecological grief in the Fernald Community Cohort: Selected and demographic variables (n = 3,737).

Variable	Frequency	Percentage		
Ecological Grief			Mean = 13.31 (SD = 3.75)	
Low	991	27.4		
Moderate	1585	43.9		
Severe	1035	28.7		
Proximity to FMPC			Mean = 3.40 (SD = 1.28)	
1 mile	429	11.5		
2 miles	1049	28.1		
3 miles	884	23.6		
4 miles	720	19.3		
5 miles	462	17.2		
6 miles	13	0.3		
Ever Diagnosed with Cancer				
Yes	682	18.2		
No	3055	81.8		
Level of Uranium Exposure				
Low	1909	53.7		
Medium	660	18.6		
High	986	27.7		
Demographic Variables				
Sex				
Female	2153	57.6		
Male	1584	42.4		
Age			Mean = 66.51 (SD = 12.61)	
38–49	404	10.8		
50–62	888	23.8		
63–75	1542	41.3	Minimum 38	Maximum 100
76–88	735	19.7		
89–100	168	4.5		
Education				
Some or less than high school	202	5.4		
High school graduate	1035	27.7		
Technical or vocational	434	9.2		
Some college	782	20.9		
College graduate	827	22.1		
Postgraduate or professional degree	504	13.5		

A factor analysis using maximum likelihood extraction with an oblique rotation showed that all items loaded on one factor, labeled Eco-grief. All items loaded at >0.70, with the Eco-grief accounting for 71% of the variance.

The model (n = 3,737) was tested with one degree of freedom, with the program variable of uranium concentration accounting for most (2.8%) of the variance, followed by ecological grief (2.1%) and ever having a cancer diagnosis (0.04%). Established criteria (CMIN; 2.00–5.00; p<0.05) for the chi-square goodness of fit index were used to determine the fit of the data to the hypothesized model [21]. The CMIN = 0.18 was not significant (p = 0.67) with one degree of freedom (CMIN/df = 0.67), which suggested an adequate fit of the data.

Established criteria (RMSEA: 0–0.10; [22]) for the root mean square error of approximation assessed differences between corresponding elements of the data to the hypothesized model. The RMSEA = 0 suggested an adequate fit of the data.

**Fig 1:**
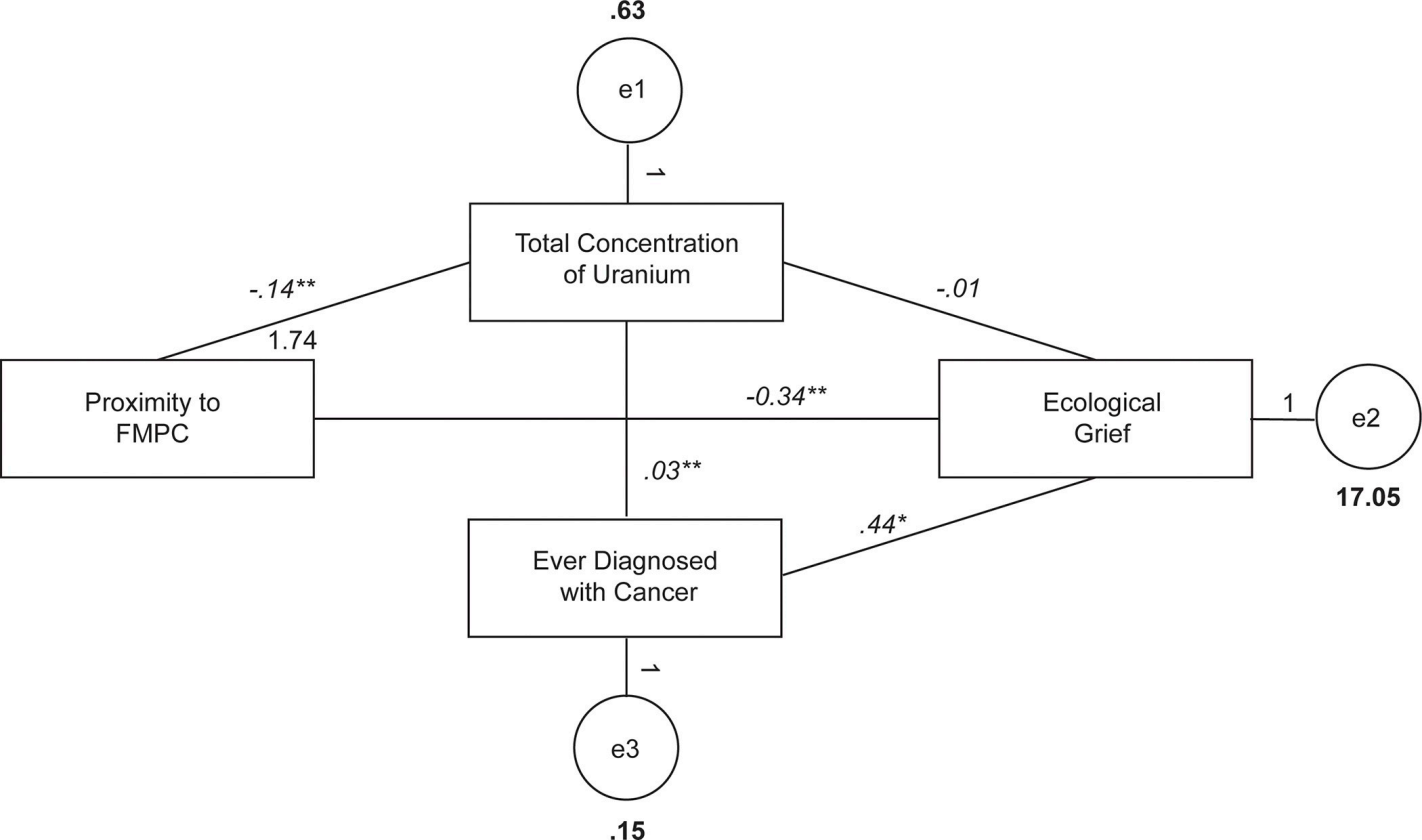
Path analysis of proximity to FMPC, total concentration of uranium, ever diagnosed with cancer and ecological grief. Variances are represented in bold values, and italicized values represent regression weights. All regression weights are significant except Total Concentration of Uranium to Ecological Grief (-.01, p = .91). Note: *p = .01, **p < .001.

Referring to Fig 1, with increased residential proximity from the FMPC (1), uranium concentration decreased (0.14; p < 0.001). With increased uranium concentration (1), cancer diagnoses increased (0.03; p < 0.001). As the living distance from the FMPC increased (1), ecological grief decreased (0.34; p < 0.001). The relationship between uranium concentration and ecological grief was not significant (p = 0.91). With increased cancer diagnoses (1), ecological grief increased (0.44; p < 0.011). Note that "(1)" is the standardized version of linear regression weights used to examine the possibility of causal linkages between statistical variables in structural equation modeling.

## Discussion

The specific aims of this cross-sectional pilot study were to 1) examine the degree of ecological grief reported by FCC participants; 2) discern the degree of ecological grief among FCC participants by proximity to the plant; 3) assess the degree of ecological grief among FCC participants by cancer diagnosis; and 4) assess the degree of ecological grief among FCC participants by level of uranium exposure. Ecological grief is especially prevalent in people with close living, working, or cultural relationships with the natural world, such as farming communities, indigenous peoples, and scientists [3,5,8,12]. It is also known that ecological loss can have direct and indirect effects on human health and safety, both physically and mentally [4,6,9,11–13]. Given that the FMPC was established in a rural farming community, which was consequently affected by environmental contamination and thus a threat to human health and safety, it was suspected that the residents of this community were impacted by ecological grief. The goal of this pilot study was to examine the phenomenon of ecological grief among FCC participants.

Specifically, it was of primary interest to examine the degree of ecological grief that the FCC participants reported while discerning the degree of grief experienced in association with residential proximity to the FMPC, cancer diagnosis, and level of uranium exposure. The results from the present study will be discussed and compared with the findings from previous research to assess consistencies or inconsistencies. Prior research has explored ecological grief due to ecological losses such as biodiversity loss, climate change, natural disasters, and extreme weather, but not yet environmental contamination. However, findings from these studies can be generalized, as they are all considered ecological losses and thus compared to assess consistencies with the findings from this pilot study.

Moderate and severe ecological grief was prevalent among 72% of respondents, meaning almost three-quarters either agreed or strongly agreed that they still get upset when they think about the environmental contamination that occurred at the FMPC, perceive that things and people in their community are still affected by the environmental contamination, believe that the environmental contamination is responsible for the loss of health or safety, or trust they have experienced and are preoccupied with thoughts about the environmental contamination that occurred. This finding is essential considering how highly prevalent ecological grief is in the sample and the fact that the sample is still experiencing ecological grief decades after the environmental contamination occurred. Researchers reported a prevalence of ecological grief, from 9% to 80%, even years after the environmental loss [6,9,11–13,23,24]. The wide range of prevalence estimates varies due to differences in the study design, sample, type of disaster, and length of investigation time post-disaster [9]. The literature has also illuminated the pronounced experience of ecological grief felt among people with close living, working, or cultural relationships with the environment [3,5,8,12]. One study was conducted in a rural farming community in Australia affected by chronic drought and wind erosion and found that the farmers felt guilt in addition to their feelings of ecological grief, blaming themselves and losing their "responsible land steward" and "good farmer" identities [2].

All participants resided within five miles of the FMPC, with an average distance of about three miles. The findings from this study showed that the further one lived or worked from the FMPC, the more ecological grief decreased. This result could be because those who live or work close to the FMPC may be more concerned about and impacted by the health consequences of environmental contamination. Previous studies have shown that people living or working in or near areas affected by ecological losses are prone to experiencing ecological grief, especially in areas at high ecological risk [2].

About 18% of the sample had a cancer diagnosis at the time of this study, and current findings showed that a cancer diagnosis was significantly associated with ecological grief. The two counties (Bulter and Montgomery) closest to the FMPC had annual cancer rates of 452.0 and 486.8 per 100,000, respectively, from 2016 to 2020. Ohio’s cancer rate per 100,000 was 465.3 from 2016–2020 [25]. Although a cancer diagnosis and its association with ecological grief have not previously been discussed in the literature, findings from this study can be compared to other physical impacts of ecological losses. This study’s results show that 35% of participants with a cancer diagnosis had severe grief compared to 21% with a cancer diagnosis who had low grief. Other studies showed that physical injury from a natural disaster was significantly associated with ecological grief [9,26]. Therefore, the present study’s finding that a cancer diagnosis was significantly associated with ecological grief could be explained by cancer’s emotionally distressing and physically harmful nature. It may also be attributed to respondent’s perception that their cancer was due to the uranium exposure they encountered.

Slightly under half (46.3%) of the sample had medium to high levels of uranium exposure. Results from data analysis found that the relationship between uranium exposure and ecological grief was not significant. Participants who resided or worked closer to the FMPC or received a cancer diagnosis were found to have higher levels of uranium exposure. Both living closer to the plant and having a cancer diagnosis were factors for experiencing ecological grief. Previous studies have suggested that ecological grief comes from direct and indirect exposure to a particular environmental loss. Direct exposure to disasters, for example, is associated with higher levels of ecological grief and can be especially traumatic for those exposed, given that most disasters put human life at risk and often involve casualties [7]. Indirect exposures can come from social media, news clips, pictures, videos, art, texts, and stories from others [27].

In summary, the results from the current study are consistent with previous research findings referred to throughout this discussion, except that a direct relationship between uranium exposure and ecological grief was not found. A high prevalence of ecological grief existed in this rural farming community. Direct relationships between ecological grief and where one lived relative to the plant and cancer diagnosis were identified, all consistent with previous findings in the literature mentioned above.

### Public health implications

Grief is a public health issue. Public health’s response to ecological grief can be carried out in various ways. Given the complexity of ecological grief, it may take various approaches to mitigate its effects on communities with high prevalence, such as the FCC. There needs to be a higher priority placed on the prevention of ecological disaster and destruction on a local, state, and federal level, as it leads to more mental, physical, and financial harm to react to ecological grief-related burdens than it would to be proactive and prevent the issue at the root cause. In addition, while discussing policies and activities that may impact the health of the environment, consideration needs to be made regarding the impacts that the degradation of environmental health has on human health since the two are intertwined. It is vital to raise awareness of what ecological grief is, how to prevent it, and how communities and individuals can cope because prolonged grief can result in maladaptive effects such as increased anxiety, depressive, and trauma-related disorders, suicide ideation, sleep disorders, substance and alcohol abuse, impairment to physical health, and disturbances in social relationships [3–7,9,11,13]. There is very little public discourse surrounding the phenomenon of ecological grief, and the current literature shows that it would be beneficial to build awareness and acknowledgment surrounding the issue. This awareness could help enhance resiliency among individuals and communities with a high prevalence of ecological grief [5]. For example, there are grief programs that help people recover from an ecological disaster. The Ohio Department of Health, through a grant to the Ohio Association of Health Commissioners, funded a grief recovery training program to prepare 25 local health district staff throughout the state to provide grief recovery services in their community, including staff in their health department. It may help people get involved in local, state, or federal organizations that are working to better the health of the environment and, thus, human health. Becoming involved in "pro-environmental" efforts and organizations has been suggested to mitigate ecological grief’s negative emotions and psychosocial effects [12]. There is no single simple solution to prevent and mitigate the effects of ecological grief. Therefore, it will take a systems approach to address this public health issue. For example, increasing knowledge of environmental risks associated with human behavior may mitigate the likelihood of future disasters attributed to human intent, negligence, or error. The findings from this research study can be used to inform and develop future interventions to mitigate the psychological impact of environmental and health loss at contamination sites. We recommend that local and state health departments train their employees in grief recovery methods.

The results of this study are consistent with other research [28–30]. These studies show that ecological grief is experienced in response to a natural or human-made disaster. The grief experienced may be increased anxiety, depression, or other manifestations. The data on ecological grief is limited; however, more researchers and community members are focused on ecological grief. One example is the academic research consortium created to work with community members, public health, and other constituencies in response to the train derailment in East Palestine, Ohio [31].

### Future directions

Ecological grief remains to be an understudied topic in the current literature. Future research in the FCC could also gather data on previous experiences of ecological grief, as the survey questions used for this pilot study only addressed current feelings of ecological grief. The potential previous experiences with ecological grief could also serve as an opportunity to learn how the respondents may have coped with or overcome their grief. Further research among the FCC participants could also study the relationship between ecological grief and its physical and psychosocial effects, as the previous literature has indicated that ecological grief is associated with a higher prevalence of anxiety, depression, and trauma-related disorders, suicide ideation, sleep disorders, and drug or alcohol abuse [3–7,9,11,13]. The previous literature has suggested that scientists and researchers such as ecologists and climate researchers are relatively susceptible to experiencing ecological grief, likely due to their close working relationship with the environment [2,5,10]. This topic could be further explored in this specific sample. It may also be worth studying the prevalence of ecological grief among non-FCC participants to examine prevalence on a much broader scale and gather a bigger-picture understanding of this phenomenon.

### Limitations

The questionnaire assessed current feelings of ecological grief, so the results cannot be used to assess any potential previous feelings of ecological grief. This study was conducted a relatively long time after the environmental contamination occurred, which may affect the results. However, this also serves as a strength of the study because it illuminates the longevity of the psychological impact that environmental contamination can have on individuals. Respondents are often biased when reporting their own experiences. For example, they may not be as truthful, unable to assess themselves accurately, their interpretation of questions can be subjective, rating scales can be considered subjective, and there is room for response and sampling bias [32]. There is also the possibility that there are unaccounted confounding factors. All of these limitations may affect the generalizability of the findings.

## Conclusions

The FCC presented a unique opportunity to study ecological grief in persons impacted by environmental loss caused by uranium contamination at the FMPC. In this study sample, it was found that ecological grief was prevalent among a significant proportion of respondents. There was a direct relationship between ecological grief and residential proximity and cancer diagnosis and an indirect relationship between ecological grief and uranium exposure. The strongest predictor of ecological grief was a cancer diagnosis. The environmental loss directly impacted the health of persons living on or near the site and challenged their long-term emotional well-being. Given the widespread emotional distress associated with ecological grief and environmental contamination’s health impact, this research’s findings warrant future studies to mitigate the psychological impact of environmental loss at the FMPC.
